# Unconscious mental imagery

**DOI:** 10.1098/rstb.2019.0689

**Published:** 2020-12-14

**Authors:** Bence Nanay

**Affiliations:** 1 Centre for Philosophical Psychology, University of Antwerp, Antwerp, Belgium

**Keywords:** mental imagery, aphantasia, priming, unconscious

## Abstract

Historically, mental imagery has been defined as an experiential state—as something necessarily conscious. But most behavioural or neuroimaging experiments on mental imagery—including the most famous ones—do not actually take the conscious experience of the subject into consideration. Further, recent research highlights that there are very few behavioural or neural differences between conscious and unconscious mental imagery. I argue that treating mental imagery as not necessarily conscious (as potentially unconscious) would bring much needed explanatory unification to mental imagery research. It would also help us to reassess some of the recent aphantasia findings inasmuch as at least some subjects with aphantasia would be best described as having unconscious mental imagery.

This article is part of the theme issue ‘Offline perception: voluntary and spontaneous perceptual experiences without matching external stimulation’.

## 1. Introduction: mental imagery

What is mental imagery? As a starting point, here is the following famous definition of mental imagery from a paper written by the all-star team of Kosslyn, Behrmann and Jeannerod:

‘Visual mental imagery is ‘seeing’ in the absence of the appropriate immediate sensory input, auditory mental imagery is ‘hearing’ in the absence of the immediate sensory input, and so on. Imagery is distinct from perception, which is the registration of physically present stimuli.**’**

[1, p. 1335]

It is not entirely clear what those quotation marks mean around the word ‘seeing’ and ‘hearing’. But the point is that, while in the case of perception the relevant mental states are brought about by the ‘the appropriate immediate sensory input’, in the case of mental imagery they are brought about without such ‘appropriate immediate sensory input’.

According to a more recent definition (without quotation marks) from a review article by Pearson *et al.* on mental imagery, ‘We use the term ‘mental imagery’ to refer to representations … of sensory information without a direct external stimulus' [2, p. 590]. And here is my own definition, which is supposed to capture the spirit of the definitions above (as well as the overall approach to mental imagery in general): mental imagery is perceptual processing (that is, processing in the early sensory cortices, V1, V2, V4/V8, MT) that is not triggered directly by sensory input [3,4].

On the Pearson *et al*. definition, mental imagery—the representation of sensory information—may be conscious or unconscious, given that representations may be conscious or unconscious. The same goes for my own definition, given that perceptual processing may also be conscious or unconscious. And while Kosslyn *et al*. are not particularly forthcoming about what ‘seeing’ in quotation marks is supposed to mean, given the theoretical commitments of all three authors about the possibility of unconscious vision and unconscious audition, if seeing and hearing can be unconscious, ‘seeing’ and ‘hearing’ (in the absence of the appropriate immediate sensory input) can presumably also be unconscious.

This way of thinking about mental imagery as perceptual processing not directly triggered by sensory input lands us with a relatively wide category that would encompass a lot of seemingly very different mental phenomena, most of which could be conscious or unconscious. All the following mental phenomena would count as mental imagery according to this definition (because they all count as early perceptual processing not triggered directly by sensory input): ‘filling in’ the blind spot, peripheral vision, amodal completion ([5,6], many (but not all)) optical illusions, many (but not all) instances of hallucination [7], dreaming, episodic memory, some perceptual expectations [8,9], phantom perception [10].

This broader category of mental imagery is useful because it gives us more explanatory unification than other, more fragmented, concepts of mental imagery would. Explanatory unification is a theoretical virtue of scientific theories [11]. The more diverse sets of findings a theory can explain the more unified it is. Considering mental imagery to be perceptual processing that is not triggered directly by sensory input gives us a highly unified way of explaining seemingly diverse mental phenomena.

We have seen that there is a strand in the current mental imagery literature that clearly allows for unconscious mental imagery (see also [12,13]). But this is very far away from being the unquestioned mainstream view. The term ‘mental imagery’ itself does not help, as it brings to mind physical images that we consciously inspect. And the philosophy of mind has consistently taken mental imagery to be necessarily conscious [14, p. 26; 15, p. 622; 16].

In the psychological and neuroscientific literature, things are a bit more complicated. Often the very same authors refer to mental imagery sometimes as a potentially unconscious representation and sometimes as a (presumably necessarily conscious) experience or even qualia (see, e.g. [17]). And some neuroscientists explicitly assume that mental imagery is necessarily conscious (see, e.g. [18]).

My aim in this paper is to settle this issue and argue that mental imagery can be unconscious. Further, I argue that treating mental imagery as not necessarily conscious (as potentially unconscious) brings much needed explanatory unification to mental imagery research. Allowing for unconscious mental imagery would also help us to reassess some of the recent aphantasia findings inasmuch as at least some subjects with aphantasia would be best described as having unconscious mental imagery.

One helpful metaphor used by neuroscientists is that of an ‘active blackboard’ [19–22]. To use the visual sense modality as an example, the general idea is that the early visual cortices (and especially V1) function as a blackboard. Various processes can write on this blackboard. Sensory—that is, retinal—stimulation automatically leaves traces on this blackboard, and does so in a retinotopic manner. To simplify a bit, what is on the retina is copied onto the blackboard.

But there are other processes that can draw on this blackboard. Mental imagery is the umbrella term for these other processes. Some of them are entirely bottom-up—determined by the sensory stimulation that shows up in other parts of the blackboard. Some others are top-down—the drawing is done by mechanisms further up in visual processing [23,24]. Some of them are voluntary processes, some involuntary. And I will try to show that while some of them are conscious, others are unconscious.

## 2. Conscious or unconscious?

Close your eyes and visualize an apple. Got it? This is undoubtedly one way of exercising our mental imagery. And one that you may consider the standard and stereotypical way of having mental imagery. But it is not at all representative.

Visualizing the apple is something you do voluntarily. But mental imagery does not have to be voluntary. One can have flashbacks of some unpleasant scene—this is also mental imagery, but it is not a voluntary exercise of mental imagery. And some of our mental imagery is of this involuntary kind—this is especially clear in the auditory sense modality, as demonstrated by the phenomenon of earworms: tunes that pop into our heads and that we keep on having auditory imagery of, even though we do not want to.

Visualizing an apple is also a conscious imaginative episode. But just as it would be a mistake to generalize from the visualizing example and claim that mental imagery is necessarily voluntary, it is also a mistake to generalize from the visualizing example and claim that mental imagery is necessarily conscious. In both cases, we would be generalizing from something that is true of some instances of mental imagery to all instances of mental imagery.

There are three kinds of reasons to think that mental imagery may be conscious or unconscious: conceptual, methodological and empirical reasons. I will spend much of the paper elaborating on the empirical reasons after touching briefly on the conceptual and the methodological reasons in this section.

First, the conceptual reason. Perception can be conscious or unconscious. If the stimulus is masked or presented for a very short period of time, the subject still perceives it, but has no conscious experience of it [25–28]. But if perception *per se* can be unconscious, it would be completely ad hoc to postulate that mental imagery cannot be. Remember that mental imagery is a form of perceptual processing: perceptual processing that is not triggered directly by sensory input. If perceptual processing that is triggered directly by sensory input can be unconscious, it is difficult to see why perceptual processing that is *not* triggered directly by sensory input (that is, mental imagery) would have to be conscious.

The second reason is methodological. Most behavioural or neuroimaging experiments on mental imagery—including the most famous ones—do not actually take the conscious experience of the subject into consideration. Take for example the famous mental rotation tasks, one of the most widely used paradigms in the study of mental imagery. There is a linear correspondence between the time required for deciding whether two three-dimensional shapes are the same and the degree of rotation between these two shapes [29]. Your task is to decide whether two complex three-dimensional shapes are the same. And you are quicker to respond (with a ‘yes’ or ‘no’ answer) if the two shapes are oriented in such a way that less mental rotation is required between them.

Whatever these experiments say about mental imagery (and we can stay away from this question), it must be a claim that is silent about whether mental imagery is conscious, as these experiments are response time experiments and the reasons for inferring the exercise of mental imagery are not introspective ones, but come from the timing of the subjects' responses (for which they did not have to be conscious of any kind of mental imagery—although they obviously needed to be conscious of the task they were performing). The mental imagery involved in this task may or may not be conscious. Therefore, the concept of mental imagery that mental rotation experiments are concerned with should not (and cannot) have consciousness as a built-in feature.

The third reason is empirical: positing unconscious mental imagery can explain a number of empirical findings better than not positing unconscious mental imagery. This is what the rest of the paper is about. My general strategy is to take the most influential arguments for unconscious perception and modify them in such a way that they are applicable to unconscious mental imagery.

Two of the most important arguments for unconscious perception come from unilateral neglect studies and priming studies. I argue in §4 and §5, respectively, that in the case of both unilateral neglect and priming, we can use the same experimental paradigm to show that not only perception, but also mental imagery, can be unconscious.

Some have recently expressed general doubt concerning the standards for when we can be absolutely certain that perception is unconscious (see [30] for a summary). These sceptics would not go along with the claim that perception can be unconscious. These worries are inherited by my arguments. If the sceptics are right that the unilateral neglect studies and the priming studies fail to establish that perception can be unconscious, then my arguments will not establish that mental imagery can be unconscious either. My last empirical argument from aphantasia (in §6) is not susceptible to these worries.

There is a third important and influential consideration for unconscious perception, namely, from dorsal vision, which could be modified to show that there is unconscious mental imagery. Without endorsing this argument, I will only sketch how it might go to then set it aside before embarking on what I take to be stronger arguments for unconscious mental imagery.

## 3. Dorsal vision

Humans (and other mammals) are thought to have two visual subsystems that use different regions of the central nervous system: the ventral and dorsal streams. To put it simply, the ventral stream is responsible for identification and recognition, whereas the function of the dorsal stream is the visual control of our motor actions. In normal circumstances, these two systems work together, but if one of them is removed or malfunctions, the other can still function relatively well (see [28,31,32], for an overview).

If the dorsal stream is malfunctioning, the agent can recognize the objects in front of her, but is incapable of manipulating them or even localizing them in her egocentric space (especially if the perceived object falls outside the agent's fovea). This is called optic ataxia. If the ventral stream is malfunctioning, a condition called visual agnosia, the agent can perform actions with objects in front of her relatively well, but she is incapable of even guessing what these objects are.

According to the received view, the dorsal visual subsystem is (normally) unconscious, and is responsible for the perceptual guidance of our actions. The ventral visual subsystem, in contrast, is (normally) conscious, and is responsible for categorization and identification [28,32–34]. So visual agnosia patients could only rely on their unconscious perception (of, say, the orientation of the object they have to manipulate) in their (quite successful) performance of visually guided actions. This observation has been widely used as an argument for unconscious perception.

Now, just as we can distinguish between dorsal vision and ventral vision [35,36], we can also distinguish between dorsal imagery and ventral imagery (the distinction goes back to [37]). And just as dorsal perception is typically unconscious, dorsal imagery is also typically unconscious ([38], see also [39] on a related typically unconscious form of action-guiding imagery). So right there, we could have an example of unconscious mental imagery. And, presumably, visual agnosia patients' visual imagery could only be unconscious.

The problem with this argument is that the assumption that dorsal vision is through and through unconscious has been criticized both on empirical and on conceptual grounds (see for example [33,34,40]). For example, we now know that there are interactions between the two streams at various points in perceptual processing (see for example [34,41,42]).

In the context of the present paper, this assumption is especially problematic, given the overwhelming evidence that visual agnosia patients’ visual imagery is not in fact unconscious. Two different visual agnosia patients (D.F., see [43], and C.K., see [44]) could manipulate and inspect their visual imagery in spite of having severely impaired ventral vision. Given what we know about the interactions between the ventral and the dorsal stream, this should not come as a surprise and it also weakens any argument from dorsal vision concerning unconscious mental imagery.

In short, there have been serious problems with considering dorsal vision unconscious through and through, and the attempts to argue that mental imagery can be unconscious with the help of the dorsal versus ventral distinction inherit all these problems. Hence, I set aside the attempts to use the dorsal/ventral distinction to argue for unconscious mental imagery and turn to empirically more solid arguments.

## 4. Unilateral neglect

Unilateral neglect is a fairly common symptom of stroke patients, impacting these patients' awareness of visual details in the contralesional side of their visual field (see [45] and [46] for summaries). Unilateral neglect patients tend to eat only from the right side of their plate, bump into obstacles on their left, and so on (for simplicity, I lump visual neglect and visual extinction together for the purposes of this paper).

Unilateral neglect is normally described as a disorder of visual attention. The general idea is that the patients are unaware of the contralesional side of their visual field because they cannot unlock their attention from the other side of their visual field. One reason to think so is the following. If a unilateral neglect patient is unaware of the left side of her visual field, then in a line-crossing task, she will only cross the lines in the right side of her visual field. But if the task is not line crossing, but line-erasing, that is, if the visual stimulus is slowly removed from the right side of her visual field, then she will eventually erase all the lines, including the ones on the left side of her visual field [47].

A lot of studies show that unilateral neglect patients unconsciously perceive what is presented to them in the contralesional side of their visual field. Stimuli presented to the contralesional side can prime their responses [48,49] and can influence their behaviour in various other ways as well, for example, their performance in forced choice tasks ([50–53], see also [54] for a sceptical take and [55] for a summary). Note that it is taken for granted in this literature that attention is necessary for consciousness, something I am happy to go along with (see [56] for a summary, but see also [57] for a dissenting view).

Unilateral neglect is not a merely perceptual phenomenon. Many neglect patients also display similar behaviour of neglecting the contralesional side of their mental imagery. This phenomenon is often called imaginal neglect. While many perceptual neglect patients also exhibit imaginal neglect symptoms, the two phenomena are doubly dissociated: one can have imaginal neglect without perceptual neglect and *vice versa* [58,59].

Imaginal neglect patients give a much less fine-grained description of the contralesional side of visualized scenes that they know very well (they could rely on semantic information about the contralesional side to give some description, but the lack of conscious awareness of the contralesional side of the mental imagery would explain why this description is (often quite radically) less fine-grained) [60]. When visualizing a very familiar image, like the map of France (for French subjects), they report hardly any features on the contralesional side [61]. The same goes for visualizing stars in the night sky [62]. Further, when dreaming (which is widely acknowledged to be a form of exercising mental imagery), the rapid eye movements (REM) of neglect patients do not move to the contralesional side [63].

Crucially, the contralesional side of the visual cortex is activated during the exercise of mental imagery [64], in much the same way as the healthy side of the visual cortex is activated when the same stimulus was presented. This should not come as a surprise—neither perceptual nor imaginal neglect is caused by damage to the visual cortex, but rather to various higher-level, attentionally involved regions.

In other words, imaginal neglect patients have early perceptual processing in the contralesional side of the visual cortex that is not triggered directly by sensory input. And this early perceptual processing is entirely unconscious. They have unconscious mental imagery.

## 5. Priming

An important set of findings that show that perception can be unconscious involve unconscious priming. The general structure of the argument here is that we can infer that the subject perceived something unconsciously if (i) the subject has no conscious awareness of the stimulus presented perceptually to her and (ii) this unconscious presentation of the stimulus primes her (often in very similar ways to that the conscious presentation of the stimulus does).

There is a large number of findings that follow this general pattern when it comes to showing that perception can be unconscious (see §2 for references). But the same general argument could be modified to show that mental imagery can be unconscious. In this case, we could infer that the subject has unconscious mental imagery if (i) the subject has no conscious awareness of her mental imagery and (ii) this unconscious mental imagery primes her in the same way conscious mental imagery does.

We have seen the general complications with (i) in §2. But in the case of unconscious mental imagery, (ii) is also more complicated than it looks. In the case of unconscious perceptual priming, as long as the subject's behaviour is altered by the unconsciously presented stimulus the same way as it is altered by consciously seeing the stimulus, we can conclude that it was her unconscious perception that primed her behaviour. But in the case of mental imagery, it is much more difficult to find behaviour that would be primed by mental imagery, let alone unconscious mental imagery.

Here is one experiment that could provide all the ingredients for the kind of argument I outlined above [65]. It is a binocular rivalry experiment. In the case of binocular rivalry, when different images are presented to the two eyes, our visual experience alternates between these two images.

Short-term exposure to stimuli immediately before the binocular rivalry task influences the pattern of these alternating experiences (as long as the stimuli are not too strong, which leads to suppression, see [66]). Suppose that an image of red vertical lines is presented to the right eye and an image of green horizontal lines is presented to the left eye. If you have been staring at the red wall before this task, your right eye (where red vertical lines are presented) is more likely to win out in the binocular rivalry—more than 50% of the time, you will experience the red vertical lines and not the green horizontal ones during the binocular rivalry task.

A relatively new set of findings show that conscious mental imagery influences the patterns of these alternating experiences in much the same way as conscious perception does [67–69]. Again, suppose that an image of red vertical lines is presented to the right eye and an image of green horizontal lines is presented to the left eye. If you visualized a red apple before the binocular rivalry phase, your right eye (where red vertical lines are presented) is more likely to win out in the binocular rivalry.

That is for conscious mental imagery. So conscious mental imagery has an impact on the binocular rivalry pattern. The question is whether unconscious mental imagery has a similar impact. Consider the following experiment [65]. Subjects were shown a two-word description of an object on the screen for 3 s, which included either the word ‘red’ or the word ‘green’ (‘red apple’, ‘red chilli’, ‘green apple’, etc.). After this, they were instructed either to imagine or to avoid imagining the described object (say, the red apple). This was followed by a 7 s period when the subjects were supposed to imagine or avoid imagining this object. In the ‘avoid imagining’ condition, if the subject did in fact, in spite of the instructions, imagine a red apple (or anything red), they had to push a button indicating this.

**Figure 1.**
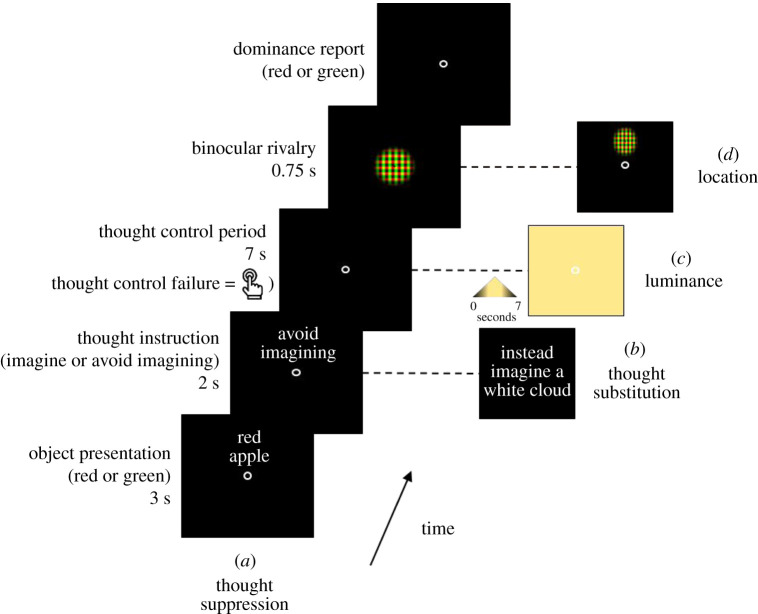
Overview of the thought suppression binocular rivalry experiment. Adapted from [65] (with permission from the author). (Online version in colour.)

After this priming phase, the subjects did the classic binocular rivalry task, with red stimulus presented to one of the eyes and green to the other and the subjects had to report which colour was dominant. A number of control conditions were added, the most important of which was identical to the ‘avoid imagining’ condition, with the exception that during the 7 s when the subject was supposed to avoid imagining, a highly luminous (neither green nor red but neutral yellow) stimulus was presented in the subject's visual field (figure 1).

The experimenters found that subjects' binocular rivalry pattern was primed just as much in the ‘avoiding imagining’ condition as in the ‘imagining’ condition. When the subjects imagined a red apple for 7 s, the red experience won out systematically in their subsequent binocular rivalry task. No surprise here. What is more surprising is that even when the subjects were avoiding imagining a red apple (and, again, ruling out those cases when they failed to avoid this) for 7 s, the red experience still won out systematically in their subsequent binocular rivalry task.

This result in itself does not rule out the possibility that what primed the binocular rivalry pattern was not unconscious mental imagery, but rather some sort of higher-level representation—maybe the linguistic representation presented on the screen (red apple) or some other cognitive strategy (see [70] on the diversity of cognitive strategies in visual working memory tasks, for example). In order to rule out this possibility, the experimenters added the control condition where during the 7 s ‘avoid imagining’ phase, the subjects were presented with a highly luminous (neither green nor red but neutral yellow) stimulus. If the priming were really due to non-sensory (say, linguistic) processes, then this should not make a difference. But it did. In the luminance condition, avoiding imagining failed to produce the same priming effect as avoiding imagining without the luminance manipulation did (or as straight imagining did).

These results strongly indicate that it is unconscious mental imagery that primes the binocular rivalry pattern. Remember that subjects had to indicate if their attempt to avoid imagining a red apple broke down. So we know that those subjects whose attempt to avoid imagining a red apple did not break down had no awareness of any red mental imagery during the 7 s period. This unconscious episode nonetheless produced the same priming effect as the conscious one did. Finally, we know that this unconscious episode was in fact unconscious mental imagery (and not some kind of unconscious higher-level (maybe linguistic) representation) given that sensory presentation of an irrelevant sensory stimulus interfered with the priming effect.

The authors of this study did not explicitly draw the conclusion that the experiment demonstrates the presence of unconscious mental imagery, but at least one of the authors of the study would be open to this interpretation (see [17], but see also [71]).

## 6. Aphantasia

All my arguments for unconscious mental imagery in the previous sections piggybacked on argumentative strategies about unconscious perception. Sceptics about unconscious perception in general would be equally sceptical about these arguments. The argument I will give in this section does not rely on any arguments about unconscious perception. It is about subjects with aphantasia.

There is a recent body of research on subjects who report not having any conscious mental imagery whatsoever. This condition is called aphantasia [72–74]. A surprisingly large proportion of the population (according to some measures 5–8%) have this condition: they lack conscious mental imagery. While it would of course be possible to argue that subjects with aphantasia are not *really* unaware of their mental imagery, this may be a more difficult (in any case, different) move compared with arguing against unconscious priming or unconscious perception in unilateral neglect. So this line of argument may convince some sceptics who are not convinced by any arguments concerning unconscious perception.

A subject has aphantasia when she reports not having mental imagery—this is a behavioural category. And, as a result, aphantasia may have a number of diverse underlying conditions. Some subjects with aphantasia have difficulties voluntarily conjuring up mental imagery, but they do report mental imagery when dreaming, for example. Some others report no mental imagery at all—either voluntary or involuntary [75].

In other words, aphantasia is not a monolithic category. Some aphantasics dream vivid dreams, clearly involving conscious visual imagery [76]. But they have problems with the control of conscious visual imagery. Others have no conscious visual imagery at all. My claim is that at least in some cases of aphantasia, we can explain the behaviour of the subjects better if we postulate that they have unconscious mental imagery. At least some aphantasics do have mental imagery, but they do not have conscious mental imagery.

One experiment [77] that very much supports my claim has a very low *N*: 1. This one subject, AI (not actual initials), is a 31-year-old female PhD student, who scored 16 points on the Vividness of Visual Imagery Questionnaire (that is the lowest possible score—the average score of the control group was 61.1 points). She reports not having any mental imagery whatsoever.

**Figure 2.**
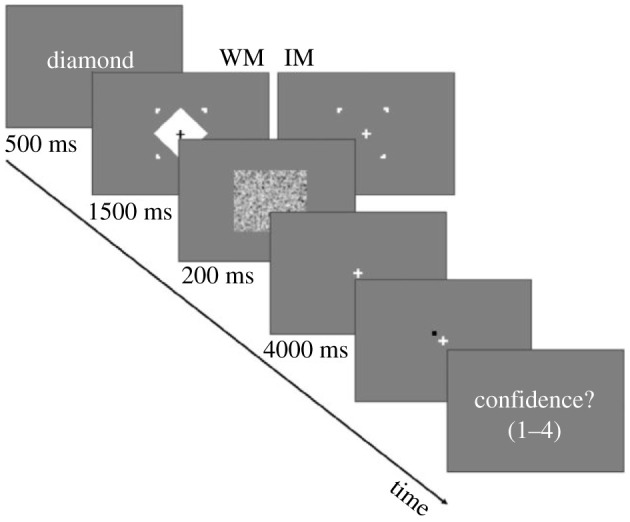
Overview of the imagining versus working memory experiment. WM, working memory; IM, imagery. Adapted from [77] (with permission from the author).

The experimental design was the following (figure 2): the subject first saw the name of a geometric shape (for example, ‘triangle’ or ‘diamond’) for 500 ms. Then she either saw the geometric shape in question framed by four placeholders for 1500 ms or was instructed to imagine this geometric shape within the four placeholders. In the latter condition, only the four placeholders were shown—four dots indicating the corners of the square within which the geometric shape was to be imagined. This was followed by 200 ms of random noise stimulus to mask the potential afterimages.

After 4 s of delay, a random dot was presented, and the subject had to decide whether it was within or without the boundaries of the perceived/imagined geometrical shape. This was followed by a confidence rating of this judgement. The only difference between the first (working memory) condition and the second (mental imagery) condition was that in the latter, the geometrical shape was not seen, but merely imagined within the region indicated by the four placeholders.

The striking finding is that the performance of AI, a subject with aphantasia, was not significantly different from controls on either of these tasks. Controls performed with a 90% success rate on the working memory task and with a 89% success rate on the mental imagery task. AI performed just around 3% worse than the controls, which is a statistically insignificant difference. AI's confidence ratings were also very similar to those of the control subjects (and generally quite high, between 3 and 4 on a 1–4 scale). The authors' conclusion is that AI's aphantasia did not have a statistically significant effect on the performance of either of these tasks.

Let us set the working memory task aside. How could we explain the finding that AI's performance on the mental imagery task was not significantly worse than the controls' performance? The straightforward explanation is that AI does use mental imagery and uses it in a very similar way to the control subjects when performing the mental imagery task. But while the controls use conscious mental imagery, AI uses unconscious mental imagery.

So far, evidence seems to support the claim that at least some subjects with aphantasia have unconscious mental imagery. But another piece of experimental finding, on the face of it, seems to go against the existence of unconscious mental imagery among aphantasics. This experiment [78], like the experiment I discussed in §5 also uses the binocular rivalry paradigm. Again, we know that conscious mental imagery influences the patterns of binocular rivalry. The question is how this process unfolds among aphantasics.

**Figure 3.**
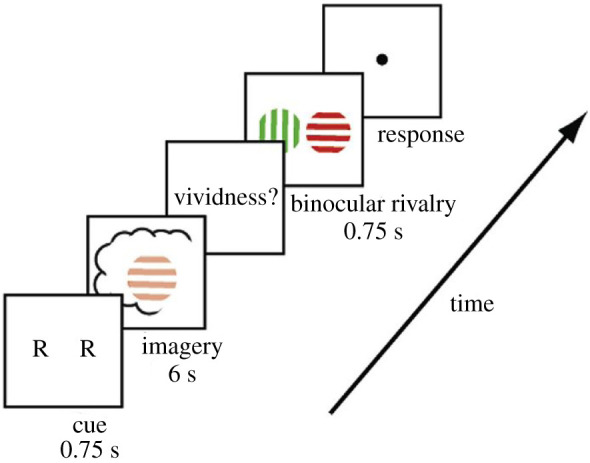
Overview of the imaginatively triggered binocular rivalry experiment from [78] (reprinted with permission from the authors). (Online version in colour.)

Participants were first taught that upon the presentation of the letter G, they are supposed to imagine green vertical lines and upon the presentation of the letter R, they should imagine red horizontal lines. During the experiment, they were shown one of these letters, which cued them to imagine either red horizontal or green vertical lines for 6 s. After this, they rated how vivid their imagery of the lines was. Finally, this was followed by the binocular rivalry task with red horizontal lines presented to one eye and green vertical lines presented to the other (figure 3).

There was no statistically significant effect of the imagining task on the binocular rivalry performance of subjects with aphantasia. While imagining red lines in the case of control subjects led to the dominance of the red lines in the binocular rivalry, in the case of subjects with aphantasia, this effect was missing.

There was another difference between aphantasics and control subjects. In control subjects, the priming effect was significantly weakened, when during the imagery phase a luminous (neither red, nor green, but neutral yellow) stimulus was shown. But in aphantasics, the luminous stimulus made no difference (it also made no difference to the invariably low ratings of the vividness of their mental imagery).

On the face of it, these findings may seem to show that aphantasics do not have any imagery, conscious or unconscious. If they had unconscious mental imagery, it would have primed the binocular rivalry performance. But it did not. One could object that, as we have seen, not all aphantasics have unconscious mental imagery, so maybe the subjects in this study do not. However, given that there were 15 subjects, all of whom showed the same response pattern, this is not a very satisfying response.

At this point, it is helpful, however, to compare these results with the [65] results I discussed in §5 above (which was conducted by the same group of researchers). I used the [65] study to show that unconscious mental imagery primes the binocular rivalry performance. So if, as I argued above, at least some aphantasics have unconscious mental imagery, their unconscious mental imagery should also prime the binocular rivalry performance. But, as the [78] experiment shows, this is not the case.

In response to this objection, a crucial difference between the two experimental setups needs to be pointed out. The mental imagery that was supposed to be triggered in the [78] experiment is voluntary mental imagery. The subjects are asked to visualize a certain stimulus, they count to three and they voluntarily try to conjure up the mental imagery. In the [65] study, in contrast, the unconscious mental imagery is involuntarily triggered. In fact, the subjects are trying not to have any imagery—they voluntarily suppress any conscious mental imagery.

So the only conclusion we can draw from the [78] experiment concerning the mental imagery of subjects with aphantasia is that their *voluntary* mental imagery does not prime their binocular rivalry performance. This says nothing about the possibility that involuntary unconscious mental imagery (in aphantasics or control subjects) would or could prime binocular rivalry performance. And, as the [65] study shows, involuntary unconscious mental imagery, at least in non-aphantasic subjects, does prime binocular rivalry performance—so nothing excludes the possibility that it does so also in subjects with aphantasia.

A lot more experimental studies could and should be done on subjects with aphantasia that could convince us conclusively that at least some aphantasics do have unconscious mental imagery. Very few neuroimaging studies have been done on aphantasics. And given the non-monolithic nature of aphantasia, we should expect a variety of different results here. But the hypothesis that some subjects with aphantasia have unconscious mental imagery could be very easily confirmed.

The studies I have focused on show that unconscious mental imagery in aphantasics and conscious mental imagery in control subjects have the same behavioural profile when the subject perform a certain task. But do they have the same neural profile? What do the visual cortices of the subject AI do in the [65] experiment? Given that we can decode the contents of visual imagery from the activation of V1 and V2 (see, e.g. [79]), it would be relatively easy to check whether AI in fact had a retinotopic representation of a diamond or a triangle in V1 and V2. If so, then it would be very difficult to argue that she does not have unconscious mental imagery.

## 7. Conclusion

Perception can be conscious or unconscious. Attention can also be conscious or unconscious. So can emotions. And there are more commonalities between conscious and unconscious perception/attention/emotions than dissimilarities. One of the aims of cognitive science (and also of the philosophy of mind) is to use categories that help us find out more about the mind. Perception (conscious or unconscious) is an explanatorily more powerful category than conscious perception. And the same goes for mental imagery. Cognitive science and especially philosophy of mind should stop obsessing over consciousness.

## Data accessibility

This article has no additional data.
